# Comparing the placement of a left-sided double-lumen tube via fiberoptic bronchoscopy guidance versus conventional intubation using a Macintosh laryngoscope, to reduce the incidence of malpositioning: study protocol for a randomized controlled pilot trial

**DOI:** 10.1186/s13063-018-3163-9

**Published:** 2019-01-15

**Authors:** Taeha Ryu, Eugene Kim, Jong Hae Kim, Seong Jun Woo, Woon Seok Roh, Sung Hye Byun

**Affiliations:** Department of Anesthesiology and Pain Medicine, School of Medicine, Catholic University of Daegu, 33, Duryugongwon-ro 17-gil, Nam-gu, Daegu, 42472 Republic of Korea

**Keywords:** Double-lumen endotracheal tube, Fiberoptic bronchoscope, Intubation, One-lung ventilation, Thoracic surgery

## Abstract

**Background:**

A fiberoptic bronchoscope (FOB) is commonly used to identify the proper placement of a double-lumen endotracheal tube (DLT) for good lung isolation during thoracic surgery. We hypothesized that the FOB-guided method for DLT placement composed of tracheal intubation under initial guidance by a FOB via the bronchial lumen and subsequent selective left-bronchial intubation could be used to reduce the incidence of DLT malposition and reduce the time required for completion of DLT placement and confirmation of proper DLT position during intubation using a left-sided DLT, in comparison to the conventional method under direct laryngoscopy using a Macintosh laryngoscope.

**Methods/design:**

In this randomized controlled pilot trial, 50 patients, aged 18–70 years, scheduled for elective thoracic surgery will be recruited and randomly assigned to two groups according to the method of DLT placement: a FOB-guided method (F) group and a conventional method (C) group. Regardless of the group, the DLT placement processes will be followed by subsequent confirmation processes, using a FOB. If the DLT is misplaced, the position would be corrected. The primary outcome is the incidence of DLT malpositioning observed via a FOB during confirmation after DLT placement. The secondary outcomes consist of the time required to achieve the entire DLT intubation process, which is the sum of the duration of DLT placement and the duration of confirmation of the proper position, the incidence of failed tracheal intubation on the first and second attempt, and complications associated with the intubation process.

**Discussion:**

This pilot study was designed as the first randomized controlled trial to confirm our hypothesis. This should provide information for a further full-scale trial, and the outcomes of the study should provide clinical evidence on the usefulness of the FOB-guided method for DLT placement, in comparison to the conventional method.

**Trial registration:**

Clinical Research Information Service; CRIS, ID: KCT0002663. Retrospectively registered on 24 January 2018.

**Electronic supplementary material:**

The online version of this article (10.1186/s13063-018-3163-9) contains supplementary material, which is available to authorized users.

## Background

Intubation using a double-lumen endotracheal tube (DLT) is essential for thoracic surgery that requires one-lung ventilation. Because of the anatomical features, i.e., that the left mainstem bronchus (LMB; average length: 5 cm) is usually longer than the right mainstem bronchus (RMB; average length: 2 cm) [1], left-sided DLTs, which should be located within the LMB, have been preferred [2]. Despite the wide safety margin of left-sided DLTs, the LMB is slightly narrower and lies in a more horizontal plane than the RMB, thereby making the optimal placement of a left-sided DLT into the LMB more difficult. Furthermore, malpositioning of the DLT even within the LMB often results in herniation of the bronchial cuff over the carina or advancement beyond the secondary carina. Therefore, identifying proper placement of the DLT after tracheal intubation is imperative to ensure appropriate function of the DLT. It is known that clinical evaluation, by inspection and auscultation, alone is unreliable for confirming the proper position of the DLT [3, 4], and consequently visual confirmation guided by fiberoptic bronchoscope (FOB) is generally considered as the gold standard [2, 5].

There are only two reported studies that have investigated the effectiveness of FOB guidance for DLT placement during intubation, as compared to the conventional intubation method [6, 7]. In both studies, even the FOB-guided technique also involved using conventional direct laryngoscopy while the DLT was inserted into the trachea, and FOB guidance was only used when advancing the DLT into the LMB. Unfortunately, Boucek et al. showed that more time was required for placement of the DLT and confirmation of its appropriate position using the FOB-guided method, than when using the conventional method [6]. In the FOB-guided method of Cheong et al., another anesthesiologist manipulated the FOB via the tracheal lumen while the DLT was inserted into the trachea, and then the bronchial tip was advanced into the LMB until the bronchial cuff was seen just below the carina [7]. Despite this somewhat cumbersome process, the FOB-guided method was found to reduce the time required for successful intubation and clinical confirmation considerably, thereby enabling the anesthesiologist to isolate the lung quickly. Furthermore, DLT placement under FOB guidance facilitated less frequent malpositioning of the DLT compared to the conventional method, in their study [7].

We hypothesized that the FOB-guided method for DLT placement, composed of tracheal intubation under initial guidance of FOB via bronchial lumen and subsequent selective left bronchial intubation, would guarantee the definite placement of the bronchial tip of the DLT within the LMB and reduce the incidence of DLT malposition. Furthermore, this process was expected to reduce the time required to confirm and correct the position of the DLT, and would expedite the entire process of DLT intubation. To our knowledge, there is no previous randomized trial that tested our hypothesis.

## Methods/design

### Study design

**Fig. 1:**
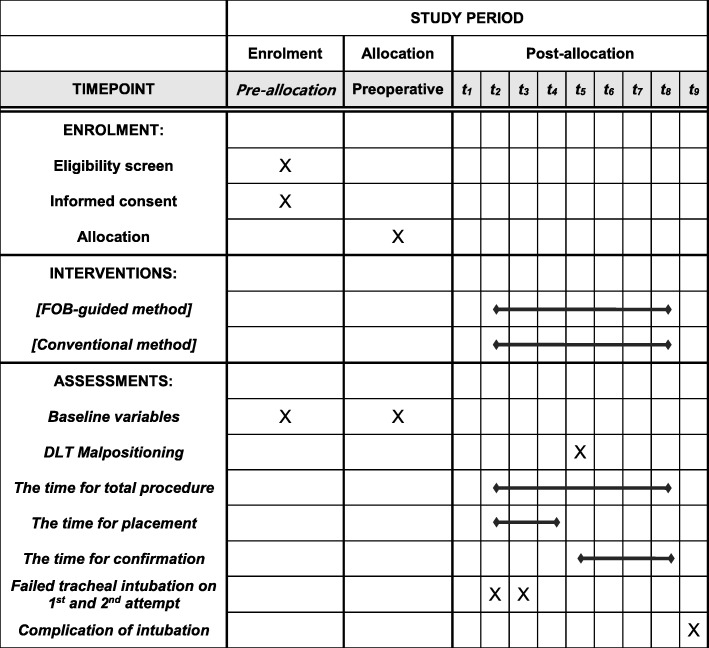
The Standard Protocol Items: Recommendations for Interventional Trials (SPIRIT) flow chart: the schedule of enrollment, interventions, and assessments. T_1_: before intubation, T_2_: DLT placement within LMB, T_3_: second attempt of DLT placement, if needed, T_4_: last attempt of DLT placement using other devices according to the discretion of the anesthesiologist, when even the second attempt fails, T_5_: confirmation of DLT position via tracheal lumen, T_6_: confirmation of DLT position via bronchial lumen, T_7_: confirmation via tracheal lumen, if applicable, T_8_: confirmation via bronchial lumen, if applicable, T_9_: after the completion of the confirmation procedure. *FOB* fiberoptic bronchoscopy, *DLT* double-lumen endotracheal tube, *LMB* left mainstem bronchus

This study is designed as a prospective, single-center, single-blind (participant), parallel- group pilot trial. The trial protocol has been approved by the Institutional Review Board of Daegu Catholic University Medical Center (CR-17-177-L) where the study will be conducted, and registered at cris.nik.go.kr (KCT0002663). The protocol of our study is described according to the Standard Protocol Items: Recommendations for Interventional Trials (SPIRIT) guidelines (see Additional file 1) and the SPIRIT flow chart (Fig. 1).

### Participants

This pilot study is designed to estimate the sample size required for a full-scale randomized controlled trial, and sample size is not calculated statistically in this pilot study. A target of 50 patients scheduled for elective thoracic surgery has been set. Potential candidates who meet the inclusion criteria will be recruited during preoperative visits, and will receive information about the study. The purpose, procedures, and potential risks and benefits of this study will be explained, and written informed consent will be obtained from each participant by the lead author (TR). It will be explained to participants that they can withdraw from the study at any time, without consequences.

Patients will be included if they meet the following criteria: (1) patients scheduled for elective thoracic surgery for which a left-sided DLT is required, (2) patients aged 18–70 years with an American Society of Anesthesiologists physical status (ASA) of I or II, and (3) patients who are willing to participate in, and comply with, the study.

Patients will be excluded if they meet one of the following criteria: (1) patients requiring a right-sided DLT, (2) patients with an intraluminal lesion in the LMB, (3) patients with an anatomical problem in the tracheobronchial tree on chest radiography, (4) patients with a body mass index (BMI) greater than 30 kg/m^2^, (5) patients with limited neck motion, (6) patients with reduced mouth opening (less than 3 cm), (7) patients with a poor dental status, and ( 8) patients with Mallampati class IV (soft palate not visible at all while sitting up straight, mouth open, and tongue maximally protruded) [8].

### Randomization and blinding

**Fig. 2:**
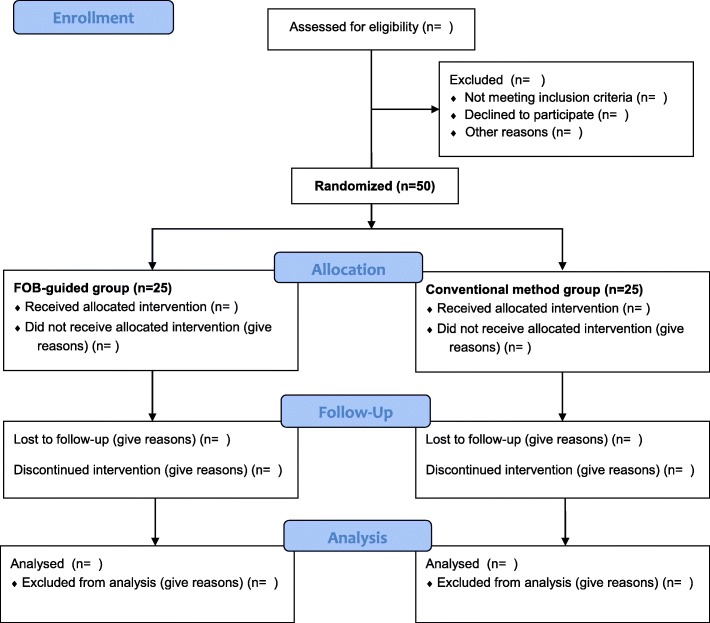
Consolidated Standards of Reporting Trials (CONSORT) flow chart

Eligible patients will be randomly assigned in equal numbers to either a conventional method (C) group or a FOB-guided method (F) group, according to the method of DLT placement (Fig. 2), using random numbers generated by Microsoft Excel 2010 (Microsoft Corp., Redmond, WA, USA), by an anesthesia nurse. The patients will be blinded to their group allocation, which will be concealed within opaque envelopes, managed by an anesthesia nurse who is not involved in the perioperative care, and opened by an anesthesiologist immediately before induction of anesthesia. However, the investigators cannot be blinded to the patients’ group allocation because there is an obvious difference between the intubation methods of the two groups.

### Anesthesia and preparation of intervention

All patients will receive midazolam (0.05 mg/kg intramuscularly) 30 min prior to induction of anesthesia. Standard monitoring, including an electrocardiogram, non-invasive blood pressure measurement, and pulse oximetry, will be applied on arrival in the operating room. A disposable bispectral index sensor (BIS™, Aspect Medical Systems, Newton, MA, USA) will be used to monitor the depth of anesthesia. Anesthesia will be induced and maintained with propofol and remifentanil using target-controlled infusion based on bispectral index monitoring of the depth of anesthesia, and 0.8 mg/kg of rocuronium will be administered for intubation. A disposable polyvinyl chloride left-sided DLT (Broncho-Cath®, Mallinckrodt Medical Ltd., Athlone, Ireland) will be used for tracheal intubation. The size of the DLT will be selected according to patients’ LMB diameters, as measured by chest computed tomographic scanning: 35 Fr for diameters < 11 mm, 37 Fr for 11 mm ≤ diameter < 12 mm, and 39 Fr for diameters ≥12 mm, based on the reports of Hannallah et al. [9]. However, the 32-Fr DLT will not be applied in this study because the FOB that can be advanced through the 32-Fr DLT would be too slender (external diameter < 3.5 mm) to use for guiding the DLT [10].

Tracheal intubation with a left-sided DLT will be performed by either the conventional method (C group) or the FOB-guided method (F group). Regardless of group assignment, the patient’s head will be placed in a sniffing position and the laryngeal view will be graded under direct laryngoscopy using a Macintosh laryngoscope according to the modified Cormack-Lehane (C/L) classification (1: full view of the glottis, 2a: partial view of the glottis, 2b: the arytenoids or posterior part of the vocal cords only just visible, 3: only the epiglottis visible, 4: neither the glottis nor the epiglottis visible) [11]. The best view obtained with or without the BURP (backward, upward, rightward pressure) maneuver will be recorded. In our study, a C/L grade 1 and 2a will be taken as denoting an “easy laryngoscopy” and grades 2b, 3, and 4 will be taken as denoting a “difficult laryngoscopy.”

### Intervention: placement of DLT and confirmation of DLT position

(1) In patients in the C group, all intubations will be performed with a DLT preformed over the stylet and the DLT will be bent to approximately 90° at the point of the tracheal balloon. After the bronchial tip of the DLT passes beyond the vocal cords under direct laryngoscopy, the stylet will be removed, and the DLT will be rotated 90° to the left and then advanced until slight resistance is encountered. Successful endotracheal intubation will be confirmed by a capnography curve. Regardless of the group, all these intubation processes, called DLT placement, will be followed by subsequent confirmation processes, performed by the same anesthesiologist.

Whether the DLT is positioned appropriately within the LMB will be confirmed using a FOB in accordance with three sequential points, as described as follow. First, when the FOB is passed through the tracheal lumen of the DLT, an unobstructed view of the RMB should be identified and the fully inflated blue-colored bronchial cuff should be positioned below the carina, without herniation. Second, the FOB will be advanced further into the RMB and an unobstructed view of right upper bronchus with the three segments should be identified. Third, when the FOB is introduced into the LMB via the bronchial lumen of the DLT, an unobstructed view of the left upper and lower bronchus should be verified.

(2) For intubation in patients in the F group, a FOB should be prepared and passed through the bronchial lumen of the DLT in advance. First, the patients’ laryngeal view will be graded according to the modified C/L classification under direct laryngoscopy [11]. Then, an anesthesiologist will introduce the FOB into the patient’s mouth while standing at the head of the bed, when a jaw-thrust maneuver will be applied to provide sufficient space for FOB passage by an experienced assistant anesthesia nurse. When positioning the FOB in the midline of the pharynx during advancement, the tip should be angulated up and down to direct it toward the glottis opening and advanced through the vocal cord [12]. Once it enters the trachea, the FOB will be advanced further into the LMB and the previously loaded DLT will be inserted into the LMB, guided by the FOB, with maintenance of jaw thrust. During withdrawal of the FOB through the bronchial lumen, the position of the DLT should be checked and corrected to ensure that the bronchial cuff is not advanced beyond the secondary carina, similarly to the third point of the conventional method described previously, while checking via the FOB. The appropriate placement is considered to be when the bronchial tip is approximately 1 cm above the secondary carina. As in the C group, after placement of the DLT, the anesthesiologist will perform the confirmation processes.

Because the third point of the conventional method during confirmation of the DLT position can be omitted in the F group, the appropriate position of the DLT within the LMB can be confirmed using a FOB in accordance with only two sequential points. First, an unobstructed view of the RMB and whether the bronchial cuff is herniated over the carina should be verified when the FOB is passed through the tracheal lumen of the DLT. At this time point, the difference from the C group is that a tube that is in a satisfactory position, without problems, will not be considered as malpositioned even when it is not in the ideal position (i.e., if the bronchial cuff is not necessarily clearly visible) because the proper position and depth of the bronchial cuff in the LMB will be confirmed via the bronchial lumen during placement of the DLT. In such a situation, the bronchial tip is likely to be in the most distal acceptable position [13]. As a second point of investigation, an unobstructed view of the right upper bronchus, along with the three segments, should be identified when the FOB is advanced further into the RMB.

### Repositioning of a malpositioned DLT and reconfirmation of position

If the DLT is determined to be malpositioned pursuant to the three points in the C group and the two points in the F group during confirmation via a FOB, it should be repositioned.

Three situations are considered as malpositions when passing the FOB via the tracheal lumen first, after placement of the DLT. In cases in which bronchial cuff herniation is discovered, the DLT should be advanced further into the LMB, and then it should be checked whether this repositioning of the DLT causes obstruction of the left upper or lower bronchus, via the opposite lumen. The other two situations involve cases in which the bronchial balloon is not visible when the FOB first passes through the tracheal lumen. In most of these cases, the DLT is likely to be inserted into the left bronchus more distally than usual, and should be withdrawn until it reaches the proper position. However, failure to find the bronchial balloon may be attributed to misplacement of the DLT into the RMB, when the tracheal lumen view is obstructed, and the carina and the distinctive orifices of the three segments of the right upper lobe cannot be recognized. In cases of suspected malpositioning into the RMB, the DLT should be withdrawn, with the FOB being passed through the bronchial lumen, until the carina can be seen normally. The FOB can then be used to direct the DLT into the LMB, as in the FOB-guided technique; it is also requisite to recheck positioning via the opposite lumen.

As this study seeks to determine which method would expedite the entire process of proper DLT intubation, the anesthesiologist performing the DLT intubation should attempt to reduce the time required to place the DLT and confirm the proper positioning of the DLT position, without wasting time unnecessarily, but also without missing key processes. The two principles of the repositioning process can be summarized as follows: When the DLT needs to be advanced more deeply into the LMB, while observing via the tracheal lumen, or withdrawn, while observing via the bronchial lumen, its position should be always rechecked through the opposite tracheal or bronchial lumen. If the DLT is repositioned while observing via the bronchial lumen (as in the FOB-guided technique), only two points should be rechecked through the opposite tracheal lumen (as in group F), even in group C.

In both groups, after placing the DLT and before confirming the position, the DLT will be fixed temporarily and mechanical ventilation will be started. After verifying the proper position of the DLT within the LMB, the DLT will be fixed firmly at the patient’s mouth using tape. The intubation and confirmation (including necessary corrections) will be performed by two experienced anesthesiologists who have more than 5 years of experience with intubation using both a conventional laryngoscope and a FOB.

### Outcome measurement

The primary outcome is the incidence of DLT malpositioning, including the herniation of the bronchial cuff over the tracheal carina, a more distal advancement than usual (advancement beyond the secondary carina in the left bronchus), or misplacement into the right bronchus, as observed via the FOB during the confirmation process after placement of the DLT.

The secondary outcomes include the measures related to reducing DLT malpositioning. Among them, the time for the total procedure is considered to reflect the effectiveness of the intubation method, and defined as the time required to achieve intubation using a left-sided DLT, which is the sum of the time for placement and for confirmation. The definitions of time durations are as follows: the time for placement is defined as the time from insertion of the laryngoscope blade or FOB tip into the patients’ mouth until removal of these devices from the mouth during DLT placement within the LMB. The time for confirmation is defined as the sum of the duration between FOB insertion and its removal through the elbow connector of the DLT during each attempt for confirmation of the proper DLT position. Up to two attempts of tracheal intubation will be allowed with the assigned technique. If a second attempt fails, the subsequent attempts will be performed at the discretion of the anesthesiologist using any other devices for successful intubation after a few minutes’ mask ventilation. The time duration of each attempt will be aggregated to determine the time for placement. The participants with failed tracheal intubation, even after two attempts, should be followed up and examined according to the intention-to-treat principle based on their assigned group. All these time points will be recorded by an assistant anesthesiologist (a resident physician).

Failed tracheal intubation on the first and second attempt (e.g., intubation into the esophagus, difficulty in inserting the DLT over the FOB into the trachea, the need for face mask oxygenation due to desaturation < 95%, or exceeding the intubation time > 90 s), and complications of the intubation process (injuries of the lip, teeth, or oropharyngeal tissues) will also be recorded as secondary outcomes.

### Withdrawal, dropout, and discontinuation

All patients will have the right to withdraw from the study at any time. Participation can be ended at any stage if the patient refuses to continue, withdraws their consent, or breaks the inclusion or exclusion criteria or the trial protocol. Participants will be withdrawn from the study if the position of the DLT cannot be verified using the FOB due to instrumental fault. The reasons for withdrawal will be recorded on the Case Report Forms.

### Confidentiality

Personal information, including the names, social security numbers, or chart numbers will not be collected. Only the study code will be collected and will be managed separately. The collected data will be kept confidential until the investigators analyze the data. After completion of the study, the collected data will be encrypted and stored for 3 years, after which it will be discarded.

### Statistical analysis

Data will be analyzed on intention-to-treat basis. Although the majority of data are expected to be collected, the mechanism and pattern of missing data will be evaluated to determine whether they have an impact on the statistical analysis and how they can be managed. The Kolmogorov-Smirnov test will be used to determine the normality of data distribution. Normally distributed data will be expressed as the mean ± standard deviation and analyzed by an independent Student’s *t* test or one-way analysis of variance. Non-normally distributed data will be given as the median (interquartile range) and analyzed using a Mann-Whitney *U* test. Categorical data will be expressed as the number of patients (percentage) and analyzed using the chi-square test or Fisher’s exact test. All comparisons are two-sided and *P* values < 0.05 will be considered to be statistically significant. Statistical analyses will be performed using IBM SPSS Statistics version 19.0.0 (IBM Corp., Armonk, NY, USA). However, sample size was not calculated statistically because this was a pilot study. There were no previous studies that could be used as a reference for sample size estimation of our study. The two previous studies mentioned above did not contain data regarding the incidence of malposition, which is the primary outcome of our study, and also their FOB-guided methods were different from our FOB-guided method [6, 7]. Therefore, we could not estimate the likely effect size for this method and required a pilot study. Although there are several guidelines for choosing an appropriate sample size for a pilot study, a recent study demonstrated that a pilot study with a size of at least 50 is advisable in many situations [14].

## Discussion

The FOB has been recommended for use as a gold standard for tracheal intubation in patients who have a difficult airway when using conventional single-lumen endotracheal tubes [15, 16], despite not being used routinely in ordinary situations. Furthermore, the FOB has generally been used in the field of thoracic anesthesia to confirm the proper positioning of the DLT [1, 2], because a well-positioned DLT allows the operative lung to collapse and provides excellent visualization of the surgical field for thoracic surgeons [5]. Therefore, use of the FOB is not unfamiliar to anesthesiologists and is an essential tool for thoracic anesthesiologists [2, 5]. If the FOB is routinely used during any thoracic surgery, the FOB-guided method, including tracheal intubation under the initial guidance of a FOB, via the bronchial lumen, and subsequent selective left bronchial intubation, may be an appropriate means for achieving intubation with a DLT, in terms of reducing the incidence of tube malpositioning.

Several papers have reported that the FOB should be used immediately after blind intubation due to the not inconsiderable rate of DLT malpositioning only detected after clinical confirmation, such as auscultation [3, 5, 17]. The incidence of misplaced DLT varies from 32 to 83%, and even in the studies reporting a lower rate (32–44%), clinical confirmation was still considered unreliable and to have missed a significant number of DLT malpositioning, which could critically affect both the patients’ safety and surgical outcomes. Not only the malpositioning per se, but also the repeated action of repositioning the misplaced tube could inflict injuries. Increased manipulation of the DLT in the bronchus may lead to excessive trauma [17]. The accuracy of initial DLT placement is likely to be improved when using a FOB for the initial guidance. Time to confirmation of correct DLT positioning by a FOB, as well as the time required to achieve the entire intubation process can be reduced because the time wasted in repositioning the DLT can be prevented using the FOB-guided method. This is beneficial in a busy operating-room setting, as it would decrease the delay in surgery preparation [17]. The precise placement of a DLT within the left bronchus under initial FOB guidance can be useful for patients as well as for medical personnel, including both anesthesiologists and surgeons. These facts form the rationale for our study and the outcomes will provide clinical evidence on the usefulness of a FOB-guided method for DLT placement, as compared with the conventional method.

This study will have a limitation, in that, the usefulness of the FOB-guided method will be limited to the patients without airway difficulty. However, the factors responsible for airway difficulty included situations wherein the intervention involving a conventional laryngoscope could also not be applied for the control group (e.g., limited mouth opening or poor dental status) or could not be used alone. For patients with a difficult airway, where the use of a conventional laryngoscopy is not feasible, an ethical issue can arise. Therefore, we wanted to compare the two methods (FOB versus conventional laryngoscope), under equivalent conditions in a situation where a conventional laryngoscope can be used. Therefore, some of the factors that could predict a difficult airway were inevitably included in the exclusion criteria, such as a BMI of 30 or higher, although this is likely to limit the generalizability of the result. Thus, for investigating the usefulness of the FOB-guided method based on the patients’ airway status in future, it is necessary to examine the patients with detailed information on the various factors associated with airway difficulty.

## Trial status

This trial is currently recruiting participants. Enrollment and trial completion is expected to be in April 2018.

## Additional file

Additional file 1: Standard Protocol Items: Recommendations for Interventional Trials (SPIRIT) 2013 Checklist: recommended items to address in a clinical trial protocol and related documents. (DOC 122 kb)

## Abbreviations

BMI: Body mass index
BURP: Backward, upward, rightward pressure
C/L: Cormack-Lehane
DLT: Double-lumen endotracheal tube
FOB: Fiberoptic bronchoscope
LMB: Left mainstem bronchus
RMB: Right mainstem bronchus
